# The reasons for not returning to work and health-related quality of life among young and middle-aged patients with stroke: A cross-sectional study

**DOI:** 10.3389/fneur.2023.1078251

**Published:** 2023-02-23

**Authors:** Xi Pan, Zhi Wang, Lin Yao, Lan Xu

**Affiliations:** ^1^Department of Neurology, The First Affiliated Hospital of Soochow University, Suzhou, Jiangsu, China; ^2^Nursing Department, The First Affiliated Hospital of Soochow University, Suzhou, Jiangsu, China

**Keywords:** young and middle-aged stroke, non-return to work, factor, quality of life, category, patient-reported outcomes

## Abstract

**Objectives:**

This study aimed to explore the reasons and influencing factors for non-return to work (non-RTW) within 1 year among young and middle-aged patients with stroke and to assess their health-related quality of life (HRQoL) at 1 year across different reasons.

**Methods:**

The study was conducted as a telephone-based cross-sectional survey. Seven hundred eighty-nine young and middle-aged patients with stroke aged between 18 and 54 years for men and 18 and 49 years for women in the electronic medical system were included. Data collection included demographic characteristics, socioeconomic status, behavioral habits, history of chronic diseases, work status, reasons for non-RTW, and HRQoL.

**Results:**

Of 789 patients, 435 (55.1%) (mean [SD] age, 47.7 [7.8] years) did not return to work within 1 year after stroke. Among the patients who did not RTW, 58.9% were unable to work, 9.7% retired early, 11.03% became full-time homemakers or were unemployed, and 20.5% were reluctant to work. The disordered multiclass logistic regression model showed that the factors influencing the reasons for non-RTW included age, gender, education, income, health insurance, diabetes comorbidity, ability to perform activities of daily living, and mobility of the right upper extremity. Furthermore, patients who were unable to work had significantly lower HRQoL compared to those who had RTW, followed by those who retired early.

**Conclusions:**

More than half did not RTW within 1 year in our study. The results will help inform future research to identify interventions to promote RTW and improve HRQoL for young and middle-aged patients with stroke.

## 1. Introduction

Recent data show that the incidence of stroke is increasing among young and middle-aged people and is highest in Asians compared to that in other ethnic groups (1, 2). According to reports, nearly 40% of patients with stroke are of working age, an age group whose specific social characteristics dictate a higher willingness to return to work (RTW) after a stroke (3). RTW is the primary goal of the rehabilitation process for most working-age patients (4), and it is closely related to the patient's quality of life, physical and mental health, subjective wellbeing, and life satisfaction (5).

Unfortunately, it can be challenging for stroke sufferers to return to work (6). Several studies have demonstrated that with proper rehabilitation, most young and middle-aged post-stroke survivors can achieve functional independence and high activity levels (1, 7). Nevertheless, the proportion of patients with stroke who do not return to work ranges from 25 to 50% (8–10). Exploring the reasons for non-RTW among young and middle-aged patients with stroke and the associated factors require clinical practice by identifying the types of non-RTW that may occur in different patients and that can be improved through rehabilitation (4, 11, 12). Although previous research has explored the factors impacting non-RTW after stroke, such as gender and advanced age (8–10), most studies have evaluated non-RTW as a whole and cannot differentiate between various non-RTW types and their associated factors. However, some qualitative studies have been conducted to explore the related causes and influencing factors (4, 11), but the researchers' opinions and thoughts may introduce bias in interpreting the results, resulting in a lack of objectivity and the inability to identify relevant influencing factors.

To the best of our knowledge, no specific study has been conducted that quantitatively describes the reason for non-RTW following stroke, and its associated factors are mainly unclear. In addition, it is uncertain whether the reported reasons for non-RTW are related to health-related quality of life (HRQoL). Therefore, the aims of this study were to (1) quantify reasons for non-RTW among young and middle-aged patients with stroke; (2) identify factors predicting different reasons for non-RTW, focusing mainly on sociodemographic and clinical characteristics factors; and (3) investigate the impact of different reasons for non-RTW on HRQoL.

## 2. Methods

The study was conducted as a telephone-based cross-sectional survey. The central review committee of the First Affiliated Hospital of Soochow University approved the study protocol (No. 2022025).

### 2.1. Participants

All patients were admitted to our neurology department between 1 July 2020 and 1 July 2021, with a diagnosis of a first-time stroke. From July 2021 to July 2022, young and middle-aged patients with stroke who had been discharged from the electronic medical system for 1 year were eligible to be surveyed by telephone. Of the 1,136 patients recorded in the electronic medical record system, 789 patients were included in this study based on the following criteria: (i) first stroke, (ii) the diagnosis of stroke (hemorrhage stroke, ischemic stroke, or hemorrhagic stroke combined with ischemic stroke), (iii) working age (18–59 years for men and 18–54 years for women) at the stroke onset, and (iv) active employment status (full-time or part-time competitive employment, or self-employment) at the stroke onset. We excluded patients who had stopped working before the onset and those with other critical illnesses, such as heart failure, respiratory failure, malignant tumors, severe trauma, and other acute diseases.

### 2.2. Data collection

A trained research assistant administered the telephone survey to participants over the phone. After obtaining verbal consent, a 20-min telephone survey was conducted.

Patient characteristics included baseline demographic characteristics (age, gender, and marital status), socioeconomic status (per capita monthly household income and education level), behavioral habits (smoking and alcohol consumption), history of comorbid chronic diseases (hypertension, diabetes, dyslipidemia, coronary heart disease, and atrial fibrillation), the stroke type, the degree of functional dependence at discharge, limb muscle strength, stroke complications (dysarthria, visual deficiency, swallowing disturbances, reduced bladder control, and sensory disturbances), and the occupational type before the onset. Among them, demographic characteristics, the history of comorbid chronic diseases, the stroke type, the degree of functional dependence at discharge, limb muscle strength, and stroke complications were obtained from the electronic medical record. Moreover, socioeconomic status, behavioral habits, and occupational type before the onset was obtained during the 20-min interview.

Age was categorized into 25–34, 35–44, and 45–55. The education level was categorized into primary school (Elementary school and below), junior high school, secondary school, or college and above. Family per capita monthly income (income level), which is equal to family income divided by the number of family members, was categorized as “ <1000,” “1001–3000,” “3001–5000,” and “>5000.” The degree of functional dependence is scored according to the activities of daily living (ADL) scale: 100 points mean no dependence, 60–99 points mean mild dependence, 40–60 points mean moderate dependence, and <40 points mean severe dependence. The muscle strength of the left upper limb, the right upper limb, the left lower limb, and the right lower limb was evaluated with a clinical examination (levels 0–5). If the muscle strength of the limb is below grade 4, the limb is considered dysfunctional.

### 2.3. Outcome

The outcome of this study was RTW after stroke, defined as active employment at the former or new occupation (full-time or part-time competitive job, or self-employment) based on these follow-up questions: (i) “Have you been able to return to work?”; (ii) “Have you changed work?”; (iii) “What is the reason for changing work?”; and (iv) “What is your reason for not returning to work?” Patients who did not return to work within 12 months were classified as non-RTW for the following reasons: (1) unable to work (if the patient reports being unable to work due to physical dysfunction), (2) early retirement (if a patient reported retiring after stroke but had not yet reached retirement age), (3) full-time homemaker or unemployed (if a patient reported becoming a full-time homemaker or being laid off after stroke), and (4) reluctance to work (if a patient reported being unable to return to work due to other reasons such as work stress, the new crown epidemic, their children's demands, or for unspecified reasons).

An additional outcome variable was health-related quality of life (HRQoL), which was measured using the health utility value of EQ-5D-5L (13), which is comprised of a five-level descriptive health classifier questionnaire and a visual analog scale (EQ-VAS). The descriptive questionnaire evaluates five dimensions (5D): mobility, self-care, usual activities, pain/discomfort, and anxiety/depression. There are five response levels (5L) for each size, ranging from no problems to extreme problems. Using the latest EQ-5D-5L health utility value conversion table based on the Chinese population, the EQ-5D-5L health status was converted into health utility values to describe the respondents' HRQoL. The health utility values range from −0.391 to 1, with zero denoting death, one representing perfect health, and negative values indicating that the current health state is worse than death. The health dimensions of the EQ-5D-5L were dichotomized into “no limitations” (“no problems”) and “limitations” (from “slight problems” to “unable”). The Cronbach's coefficient was 0.761 in the study.

### 2.4. Statistical analysis

This study used descriptive statistics such as mean, standard deviation, and frequency to describe the demographic characteristics and reasons for RTW and HRQoL variables. The chi-square test was used to compare patient characteristics between the RTW and four non-RTW groups. We fitted a disordered multiclass logistic regression model to evaluate the association between patient characteristics and the four reasons for non-RTW, with RTW as the reference. Furthermore, descriptive statistics for the EQ-5D dimensions, EQ-5D index, and EQ-VAS were calculated. Differences in the distribution of continuous variables over different categorical groups were evaluated using the Kruskal–Wallis test, and where differences were detected, Dunn's test was used for pairwise comparisons. For nominal variables, a chi-square test was used as applicable. All statistical tests were performed using a two-sided α value of 0.05. Analyses were conducted using SPSS, version 22.0.

## 3. Results

### 3.1. Baseline patient characteristics

**Figure 1:**
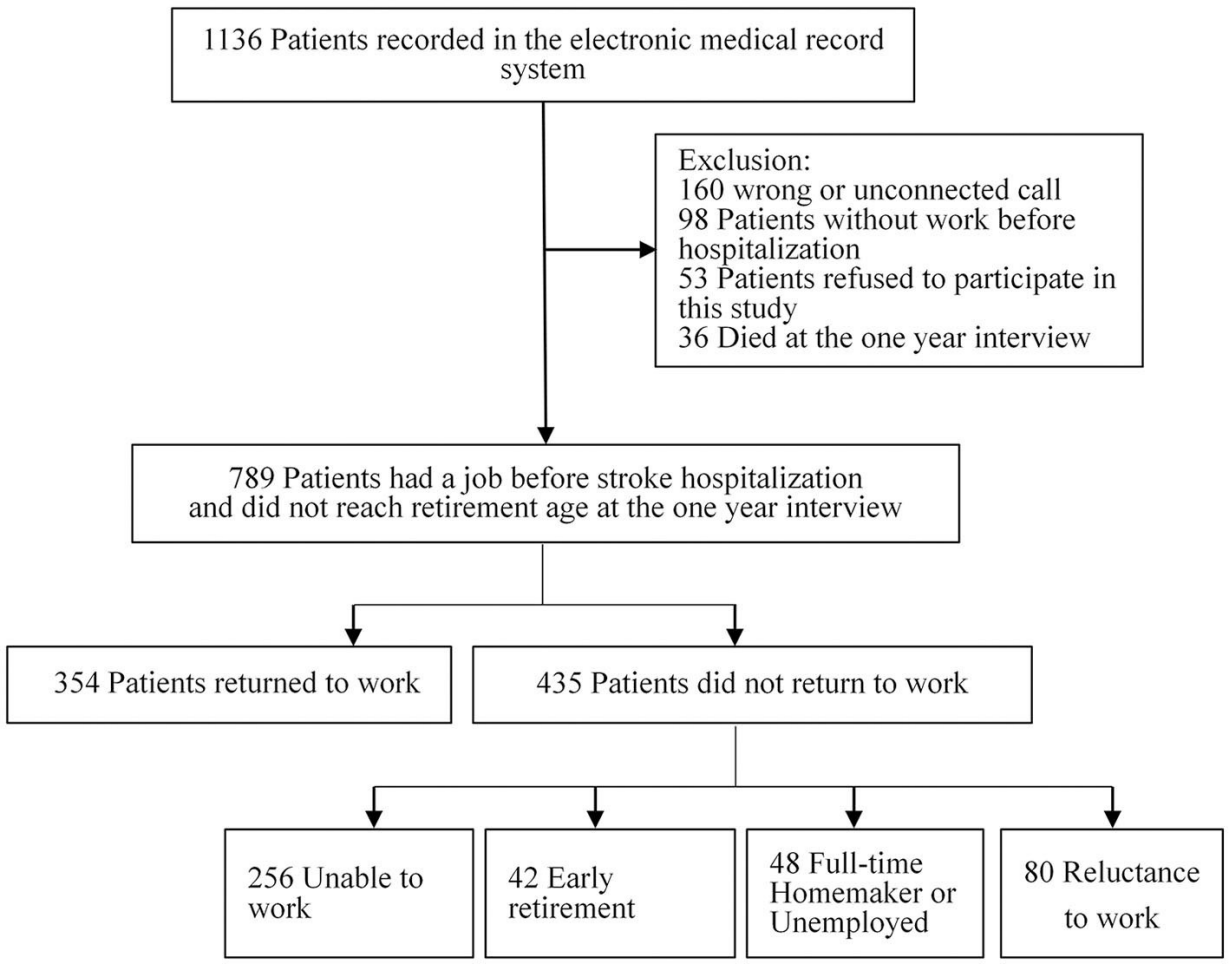
Flowchart of patient enrollment.

The final study sample included 1,136 patients in the electronic medical record system (Figure 1). Among the 789 patients, 576 (73.0%) were men, and 740 (93.8%) were patients with ischemic stroke, 153 (19.4%) had a college degree or higher, 169 (21.4%) were physical workers. The mean (SD) age of these patients was 47.68 (7.8) years; 647 (82.0%) were aged 40 years or older. The six common chronic diseases in the population were hypertension (456 [57.8%]), diabetes (160 [20.3%]), dyslipidemia (18 [2.3%]), atrial fibrillation (19 [2.4%]), coronary heart disease (17 [2.2%]), and kidney disease (13 [1.7%]). The five common dysfunctions owing to stroke were dysarthria (155 [19.7%]), visual deficiency (19 [2.4%]), dysphagia (45 [5.7%]), reduced bladder control (24 [3.0%]), and sensory disturbance (108 [13.7%]) (Table 1).

**Table 1:** Distribution and comparison of reasons for non-RTW among young and middle-aged patients with stroke.

**Variable**	**Return to work (*n =* 354)**	**Unable to work (*n =* 256)**	**Early retirement (*n =* 42)**	**Full-time homemaker or unemployed (*n =* 48)**	**Reluctance to work (*n =* 89)**	***P***
Age at onset						0.000^*^
18–30	12 (70.6)	1 (5.9)	0 (0.0)	2 (11.8)	2 (11.8)	
30–40	68 (62.4)	23 (21.1)	1 (0.9)	5 (4.6)	12 (11.0)	
40–50	150 (56.6)	77 (29.1)	3 (1.1)	16 (6.0)	19 (7.2)	
50	124 (31.2)	165 (41.5)	37 (9.3)	25 (6.3)	47 (11.8)	
Gender						0.000^*^
Male	286 (49.7)	191 (33.2)	27 (4.7)	23 (4.0)	49 (8.5)	
Female	68 (31.9)	76 (35.2)	14 (6.6)	24 (11.7)	31 (14.6)	
Education						0.000^*^
Elementary school and below	36 (28.1)	54 (42.2)	4 (3.1)	16 (12.5)	18 (14.1)	
Junior high school	122 (38.5)	131 (41.0)	19 (6.0)	19 (6.3)	26 (8.2)	
High school/secondary school	95 (54.3)	49 (28.0)	11 (6.3)	7 (4.0)	13 (7.4)	
College and above	95 (62.1)	27 (17.7)	6 (3.9)	4 (2.6)	21 (13.7)	
Family per capita monthly income						0.000^*^
<1000	5 (13.9)	22 (61.1)	0 (0.0)	5 (13.9)	4 (11.1)	
1001–3000	47 (30.7)	66 (43.1)	7 (4.6)	16 (10.5)	17 (11.1)	
3001–5000	132 (50.0)	81 (30.3)	15 (5.7)	15 (5.7)	22 (8.3)	
>5000	131 (57.7)	62 (27.3)	6 (2.6)	5 (2.2)	23 (10.1)	
Medical insurance	277 (48.2)	176 (30.6)	30 (5.2)	35 (6.1)	57 (9.9)	0.018^*^
Prior smoking	73 (52.9)	48 (34.8)	5 (3.6)	6 (4.1)	6 (4.4)	0.050
Prior drinking	45 (45.9)	42 (42.9)	4 (4.1)	1 (1.0)	6 (6.1)	0.048^*^
Stroke type						0.093
Ischemic	340 (46.0)	240 (32.3)	39 (5.3)	45 (6.2)	76 (10.3)	
Hemorrhagic	14 (30.4)	24 (52.2)	2 (4.4)	2 (4.4)	4 (8.7)	
Mixed	0 (0)	3 (100)	0 (0.0)	0 (0.0)	0 (0.0)	
**Chronic diseases**						
Hypertension	183 (40.1)	181 (39.7)	26 (5.7)	26 (5.7)	40 (8.8)	0.001^*^
Diabetes	54 (33.8)	61 (38.1)	15 (9.4)	15 (9.4)	15 (9.4)	0.002^*^
Dyslipidemia	13 (72.2)	2 (11.1)	0 (0.0)	2 (11.1)	1 (5.6)	0.090
Atrial fibrillation	9 (47.4)	7 (36.8)	1 (5.3)	1 (5.3)	1 (5.3)	0.968
Coronary heart disease	5 (29.4)	7 (41.2)	2 (11.8)	2 (11.8)	1 (5.9)	0.414
Kidney disease	5 (38.5)	6 (46.2)	1 (7.7)	0 (0.0)	1 (7.7)	0.784
Daily life dependence						0.000^*^
No dependency	246 (58.9)	84 (20.1)	23 (5.5)	23 (5.5)	42 (10.1)	
Mild dependence	71 (35.0)	84 (41.4)	9 (4.4)	16 (7.9)	23 (11.3)	
Moderate dependence	24 (35.8)	28 (40.3)	4 (6.0)	4 (7.5)	7 (10.5)	
Heavy dependence	13 (12.9)	71 (70.3)	5 (5.0)	4 (4.0)	8 (7.9)	
LU extremity dysfunction	15 (18.8)	51 (63.8)	4 (5.0)	5 (6.3)	5 (6.3)	0.000^*^
LL extremity dysfunction	9 (15.5)	38 (65.5)	3 (5.2)	3 (5.2)	5 (8.6)	0.000^*^
RU extremity dysfunction	7 (8.9)	57 (72.2)	6 (7.6)	2 (2.5)	7 (8.9)	0.000^*^
RL extremity dysfunction	4 (6.7)	48 (80.0)	3 (5.0)	2 (3.3)	3 (5.0)	0.000^*^
Dysarthria	43 (27.7)	83 (53.6)	4 (2.6)	5 (3.2)	20 (12.9)	0.000^*^
Visual deficiency	7 (36.8)	6 (31.6)	2 (10.5)	1 (5.3)	3 (15.8)	0.735
Dysphagia	8 (17.8)	33 (73.3)	0 (0.0)	2 (4.4)	2 (4.4)	0.000^*^
Reduced bladder control	3 (12.5)	20 (83.3)	1 (4.2)	0 (0.0)	0 (0.0)	0.000^*^
Sensory disturbance	40 (37.0)	44 (40.7)	3 (2.8)	7 (6.5)	14 (13.0)	0.205
Pre-stroke occupation						0.001^*^
Non-physical	102 (67.6)	30 (19.2)	5 (3.3)	2 (2.0)	12 (8.0)	
Physical	98 (58.0)	48 (28.4)	1 (0.6)	8 (4.7)	14 (8.3)	
Combination	146 (50.5)	96 (33.2)	16 (5.5)	17 (5.9)	14 (4.8)	

* *P*-value < 0.05.

### 3.2. Non-return to work

In total, 354 patients (44.9%) returned to work within 1 year after discharge from a stroke. Among them, 291 patients (36.9%) returned to their original work, 37 patients (4.7%) changed work owing to stroke, and 26 patients (3.3%) changed work for other reasons. Among the 435 patients who did not RTW, 256 (32.5%) were unable to work owing to stroke, 42 (5.2%) retired early owing to stroke, 48 (6.1%) became full-time homemakers or were unemployed, and 89 (11.3%) showed reluctance to work (Table 2).

**Table 2:** Work status within 1 year among young and middle-aged patients with stroke (*n* = 789).

**Work status**	***n =* 789**
RTW	354 (44.9)
Returned to their original work	291 (36.9)
Changed work owing to stroke	37 (4.7)
Changed work owing to other reasons	26 (3.3)
Non-RTW	435 (55.1)
Unable to work	256 (32.5)
Early retirement	42 (5.2)
Full-time homemakers or were unemployed	48 (6.1)
Reluctance to work	89 (11.3)

RTW, return to work.

### 3.3. Factors for non-return to work

**Figure 2:**
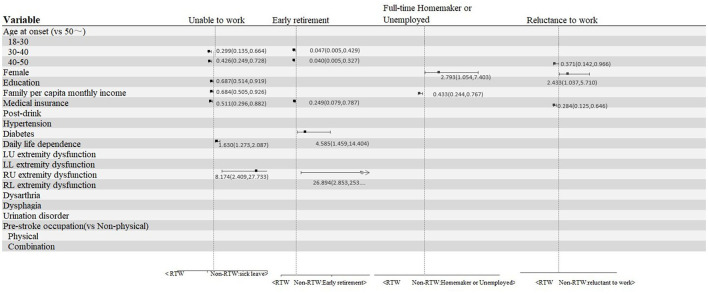
Variables statistically significantly associated with at least one of the four non-RTW reasons.

Baseline data showed that factors influencing the reason for non-RTW included age at onset, gender, education level, per capita monthly household income, medical insurance, hypertension, diabetes mellitus, daily life dependence, the muscle strength of the four limbs, and pre-stroke occupation (Table 1). Furthermore, with different reasons for non-RTW as dependent variables (with RTW as the control) and variables with statistical significance in the univariate analysis as independent variables, an unordered multiclass logistic regression analysis was performed. The multinomial logistic regression modeling results are presented in Table 3. Younger patients are less likely to be unable to work and retire earlier than older patients. Patients aged 40–50 years were less likely than those aged 50 years or older to be reluctant to work (odds ratio [OR], 0.371; 95% CI, 0.142–0.966). Female patients were more likely than male patients to be at home full time (OR, 2.793; 95% CI, 1.054–7.403) and to be reluctant to work (OR, 2.433; 95% CI, 1.037–5.710). The likelihood of being unable to work decreases as education increases (OR, 0.687; 95% CI, 0.514, 0.919). As monthly per capita household income increases, the possibility of being unable to work (OR, 0.684; 95% CI, 0.505–0.926) and being at home full time (OR, 0.433; 95% CI, 0.244–0.767) decreases. Patients with medical insurance were less likely to be unable to work (OR, 0.511; 95% CI, 0.296–0.882), to retire early (OR, 0.249; 95% CI, 0.079–0.787), and to be reluctant to work (OR, 0.284; 95% CI, 0.125–0.646) than those without medical insurance. Patients with diabetes were more likely to choose early retirement than those without diabetes (OR, 4.585; 95% CI, 1.459–14.404). The likelihood of being unable to work increases as the dependence on daily life increases (OR, 1.630; 95% CI, 1.273–2.087). Patients who cannot lift their right upper limb are more likely to be unable to work (OR, 8.174; 95% CI, 2.409–27.733) and to retire early (OR, 26.894; 95% CI, 2.853–253.551) than those who can lift their right upper limb. Dysphagia, dysarthria, dysuria, and sensory disorder after a stroke had no significant effect on the reasons for non-RTW (Table 3). Figure 2 summarizes Table 3, a visualization of the statistically significantly associated variables with at least one of the four non-RTW reasons.

**Table 3:** Factors of reasons for non-RTW: Multinomial logistic regression (vs. RTW).

	**Unable to work**		**Early retirement**		**Full-time Homemaker or Unemployed**		**Reluctance to work**	
**Variable**	**OR (95% CI)**	***P***	**OR (95% CI)**	***P***	**OR (95% CI)**	***P***	**OR (95% CI)**	***P***
**Age at onset**								
18-30	€	0.997	-	-	2.18 (0.18, 26.35)	0.540	0.00 (0.00, 0.00)	-
30-40	0.30 (0.14, 0.66)	0.003	0.05 (0.01, 0.43)	0.007	0.67 (0.15, 2.90)	0.587	0.61 (0.21, 1.77)	0.362
40-50	0.43 (0.25, 0.73)	0.002	0.04 (0.01, 0.33)	0.003	0.78 (0.28, 2.17)	0.632	0.37 (0.14, 0.97)	0.042
50	Ref	-	Ref		Ref		Ref	
Female	1.66 (0.93, 2.96)	0.089	3.11 (0.94, 10.49)	0.064	2.79 (1.05, 7.40)	0.039	2.43 (1.04, 5.71)	0.041
Education	0.69 (0.51, 0.92)	0.011	1.25 (0.67, 2.32)	0.484	0.58 (0.33, 1.04)	0.066	1.01 (0.61, 1.66)	0.974
Family per capita monthly income	0.68 (0.51, 0.93)	0.014	1.07 (0.51, 2.21)	0.864	0.43 (0.24, 0.77)	0.004	0.99 (0.59, 1.66)	0.959
Medical insurance	0.51 (0.30, 0.88)	0.016	0.25 (0.08, 0.79)	0.018	0.52 (0.19, 1.42)	0.199	0.28 (0.13, 0.65)	0.003
Prior drinking	0.93 (0.45, 1.93)	0.841	2.58 (0.55, 12.18)	0.231	0.44 (0.05, 3.75)	0.451	0.51 (0.11, 2.45)	0.397
Hypertension	1.29 (0.78, 2.11)	0.323	0.49 (0.17, 1.47)	0.204	0.91 (0.36, 2.32)	0.845	0.64 (0.29, 1.42)	0.272
Diabetes	1.25 (0.68, 2.29)	0.472	4.59 (1.46, 14.40)	0.009	1.78 (0.62, 5.19)	0.285	1.21 (0.41, 3.56)	0.731
Daily life dependence	1.63 (1.27, 2.09)	0.000	0.93 (0.43, 2.03)	0.855	1.27 (0.80, 2.03)	0.316	1.31 (0.84, 2.03)	0.232
LU extremity dysfunction	3.16 (0.94, 10.66)	0.064	0.68 (0.01, 43.00)	0.855	6.58 (0.95, 45.42)	0.056	0.57 (0.02, 13.33)	0.725
LL extremity dysfunction	0.72 (0.15, 3.51)	0.680	18.04 (0.24, 1386.92)	0.192	0.49 (0.04, 6.37)	0.585	1.62 (0.06, 44.40)	0.775
RU extremity dysfunction	8.17 (2.41, 27.73)	0.001	26.89 (2.85, 253.55)	0.004	6.83 (0.77, 60.32)	0.084	3.68 (0.44, 31.02)	0.231
RL extremity dysfunction	1.60 (0.33, 7.76)	0.561	0.00 (0.00, -)	0.997	0.86 (0.04, 18.04)	0.923	0.97 (0.06, 16.93)	0.981
Dysarthria	1.31 (0.67, 2.55)	0.427	0.50 (0.05, 4.64)	0.541	0.73 (0.16, 3.29)	0.677	1.20 (0.36, 4.01)	0.773
Dysphagia	0.78 (0.20, 3.13)	0.728	0.00 (0.00, -)	0.998	1.79 (0.18, 18.00)	0.621	0.00 (0.00, .c)	0.998
Reduced bladder control	1.70 (0.27, 10.87)	0.577	0.00 (0.00, -)	0.999	0.00 (0.00, 0.00)	-	0.00 (0.00, -)	0.998
**Pre-stroke occupation**								
Non-physical	Ref		Ref		Ref		Ref	
Physical	1.70 (0.87, 3.34)	0.120	1.96 (0.49, 7.80)	0.343	2.19 (0.54, 8.94)	0.274	0.86 (0.30, 2.46)	0.782
Combination	0.85 (0.38, 1.87)	0.683	0.11 (0.01, 1.63)	0.109	0.75 (0.14, 4.04)	0.739	0.97 (0.274, 3.40)	0.957

e: Not analyzed because cell size < 5; OR, odds ratio; CI, confidence interval; RTW, return to work.

### 3.4. Health-related quality of life in the non-RTW groups

**Figure 3:**
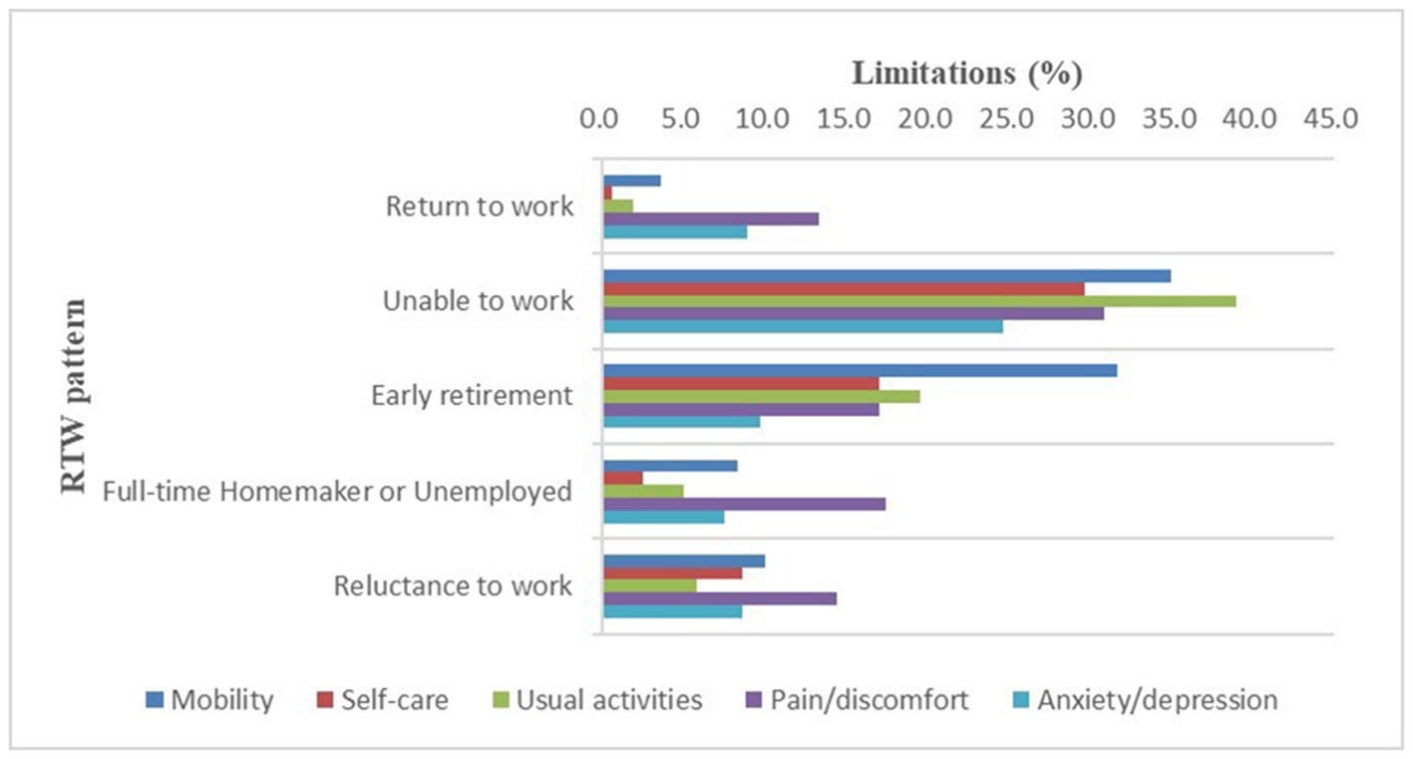
Limitations (%) per health domain of the EQ-5D-5L among patients who have returned to work and the four groups of patients who have not returned to work.

**Figure 4:**
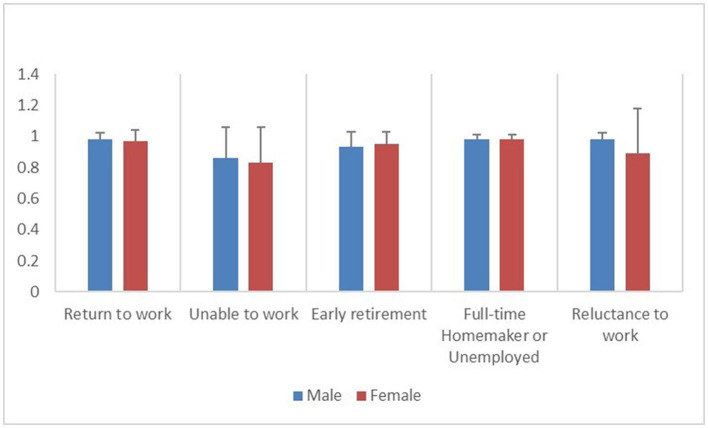
Distribution of EQ-5D index among patients who have returned to work and the four groups of patients who have not returned to work, stratified by gender.

**Figure 5:**
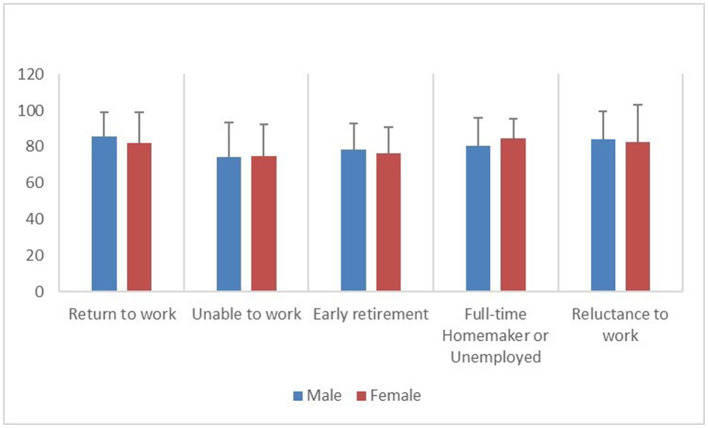
Distribution of EQ-5D VAS among patients who have returned to work and the four groups of patients who have not returned to work, stratified by gender.

The most prominent problem in the “unable to work” group was the usual activities (38.94%). The most significant problem in the “early retirement” group was mobility (31.71%). In the other three groups, including those who had returned to work, the most prominent problem was pain/discomfort (13.38, 17.50, and 14.49%), as shown in Figure 3. Compared to patients who had RTW, those who were unable to work reported higher rates of health problems in all dimensions of the EQ-5D-5L; those who retired early reported higher rates of health problems in the mobility, self-care, and usual activities dimensions (30.95, 16.67, and 19.05%); and those who were reluctant to work reported the higher rates of health problems in the self-care dimension (6.74%). When stratified by gender, male patients had similar rates of health problems as the overall population, while female patients who were unable to work had higher rates of health problems in the mobility, self-care, and usual activities dimensions (36.84, 28.95, and 36.84%). Female patients who retired early had higher rates of health problems in their usual activities (14.29%). Female patients who were reluctant to work had an increased proportion of self-care health problems (16.13%) (Table 4). Furthermore, compared to patients who had RTW, patients who were unable to work had significantly lower EQ-5D index and EQ-5D VAS (*P* < 0.05), male patients who retired early had significantly lower EQ-5D index and EQ-5D VAS (*P* < 0.05), and female patients who retired early had significantly lower EQ-5D VAS (*P* < 0.05). There was no significant difference between the female patients who were reluctant to work and those who were unable to work in terms of the EQ-5D score. Male patients who were full-time homemakers or unemployed had the second-lowest EQ-5D VAS, behind those who were unable to work and those who retired early, although the difference was not statistically significant (Table 5; Figures 4, 5).

**Table 4:** Percentage of health problems in five dimensions of EQ-5D-5L, stratified by gender [*n* (%)].

		**MO**	**SC**	**UA**	**PD**	**AD**
Total	Return to work (*n =* 354)	13 (3.7)	2 (0.6)	6 (1.7)	42 (11.9)	28 (7.9)
(*n =* 789)	Unable to work (*n =* 256)	93 (36.3)^*^	67 (26.2)^*^	88 (34.4)^*^	70 (27.4)^*^	56 (21.9)^*^
	Early retirement (*n =* 42)	13 (31.0)^*^	7 (16.7)^*^	8 (19.1)^*^	7 (16.7)	4 (9.5)
	Full-time Homemaker or Unemployed (*n =* 48)	4 (8.3)	1 (2.1)	2 (4.2)	7 (14.6)	3 (6.3)
	Reluctance to work (*n =* 89)	8 (9.0)	6 (6.7)^*^	4 (4.5)	10 (11.2)	6 (6.7)
Male	Return to work (*n =* 286)	11 (3.9)	2 (0.7)	6 (2.1)	28 (9.8)	21 (7.3)
(*n =* 576)	Unable to work (*n =* 191)	65 (34.0)^*^	45 (23.6)^*^	60 (31.4)^*^	46 (24.2)^*^	41 (21.5)^*^
	Early retirement (*n =* 27)	10 (37.0)^*^	6 (22.2)^*^	6 (22.2)^*^	4 (14.8)	2 (7.4)
	Full-time Homemaker or Unemployed (*n =* 23)	1 (4.4)	0 (0.0)	0 (0.0)	3 (13.0)	1 (4.4)
	Reluctance to work (*n =* 49)	4 (8.2)	1 (2.0)	1 (2.0)	4 (8.2)	1 (2.0)
Female	Return to work (*n =* 68)	2 (2.9)	0 (0.0)	0 (0.0)	14 (20.6)	7 (10.3)
(*n =* 213)	Unable to work (*n =* 76)	28 (36.8)^*^	22 (29.0)^*^	28 (36.8)^*^	24 (31.6)	15 (19.7)
	Early retirement (*n =* 14)	3 (21.4)	1 (7.1)	2 (14.3)^*^	3 (21.4)	2 (14.3)
	Full-time Homemaker or Unemployed (*n =* 24)	3 (12.5)	1 (4.2)	2 (8.3)	4 (16.7)	2 (8.3)
	Reluctance to work (*n =* 31)	4 (12.9)	5 (16.1)^*^	3 (9.7)	6 (19.4)	5 (16.1)

* Comparisons to return to work group, *P*-value < 0.05. MO, mobility; SC, self-care; UA, usual activities; PD, pain/discomfort; AD, anxiety/depression.

**Table 5:** Health-related quality of life across different reasons for non-RTW, stratified by gender.

	**EQ-5D index**			**EQ-5D VAS**		
	**Total**	**Male**	**Female**	**Total**	**Male**	**Female**
Return to work	0.98 ± 0.5	0.98 ± 0.1	0.97 ± 0.1	85.0 ± 14.0	85.7 ± 13.1	81.8 ± 17.2
Unable to work	0.86 ± 0.2^*^	0.86 ± 0.2^*^	0.83 ± 0.2^*^	74.6 ± 18.4^*^	74.4 ± 18.8^*^	75.0 ± 17.3^*^
Early retirement	0.94 ± 0.1	0.93 ± 0.1^*^	0.95 ± 0.1	77.7 ± 14.2^*^	78.3 ± 14.3^*^	76.4 ± 14.5
Full-time Homemaker or Unemployed	0.98 ± 0.1	0.98 ± 0.1	0.98 ± 0.0	82.8 ± 13.1	80.5 ± 15.5	84.6 ± 11.0
Reluctance to work	0.95 ± 0.2	0.98 ± 0.1	0.89 ± 0.3	83.4 ± 17.6	84.0 ± 15.6	82.5 ± 20.6

* Comparisons to return to the work group, *P*-value < 0.05.

## 4. Discussion

We found that more than half of previously employed individuals did not return to work within 1 year of being hospitalized for a stroke. Among those who were non-RTW, 32.45% were unable to work due to health reasons, 5.23% retired early, 6.08% were full-time homemakers or were unemployed, and 11.28% were reluctant to work. Moreover, our study explored various demographic, socioeconomic, and clinical factors associated with reasons for non-RTW, which the association may be informative when planning interventions for recovery after stroke. Furthermore, the HRQoL of patients who were unable to work was significantly lower than those who had RTW, followed by those who retired early. In addition, female patients who were reluctant to work had a lower EQ-5D index second only to those who were unable to work, which may be related to a higher rate of limitations with self-care. Similarly, male patients who were unable to work, retired early, and stayed at home full time had lower EQ-5D VAS.

In the present study, <50% of patients with stroke returned to work within 1 year after discharge from the hospital. This rate is relatively lower compared to other countries, where rates have ranged between 50 and 75% over the past two decades (8–10). Several factors may explain this observation. First, the accessibility of post-stroke rehabilitation services in China is poor, and the vocational rehabilitation system is not well developed (14). Vocational rehabilitation can effectively facilitate the RTW of patients with stroke, improving their mood, physical function, participation, health-related quality of life, work self-efficacy, and confidence (15, 16). Second, the age-based retirement policy implemented in the country could have a role. Currently, men retire at 60 and women at 55. Given that 50.44% of the study participants were 50 and over, pension policies hampered RTW motivation, especially for women whose retirement age was 5 years younger. Previous studies have shown that the rate of RTW after stroke varies within and between countries. For example, the rate is 59% to 68% in the United States (10, 17), 65% to 74% in Sweden (8, 18, 19), 70% in Israel (20), 75% in Germany (21), 75% in Finland (22), 72% in the Netherlands (23), 50% in Denmark (9), and 55% in Japan (24). Across countries, there may be differences in sampling practices, current unemployment rates, sickness benefits, insurance assistance, social assistance programs, or employment protection laws.

We identified several sociodemographic and clinical characteristics associated with reasons for non-RTW. Many studies have reported that daily life dependence and right upper limb paralysis after stroke adversely affect RTW (23, 25). In particular, the right upper extremity hand function is essential in early rehab, as it directly affects the ability to work. However, the corresponding confidence intervals are wide, making it impossible to determine the true effect. Similar results suggest that socioeconomic levels, such as age, education level, income, and medical insurance, may be an additional important factor in determining RTW. This result is consistent with earlier Swedish and international studies (8). Meanwhile, patients aged 40–50 years were 0.629 times less likely to be reluctant to work than those aged 50 or older. This could be because middle-aged patients in this age group bear the financial burden of supporting their parents and children simultaneously, and traditional Chinese culture dictates that they are less likely to be unwilling to work when they are able to do so. Furthermore, people with diabetes were more likely to choose early retirement. Patients with diabetes have to consistently consider their diet, exercise, medication, and blood glucose monitoring in their job routines, which can have a detrimental effect on their treatment and make managing the disease even more complicated, potentially leading to early retirement.

More importantly, we report the HRQoL associated with non-RTW attributed to different reasons. Patients unable to work had the lowest 1-year health-related quality of life, which was related to the effects of stroke. Moreover, patients who were unable to work had the highest rates of health problems in all five dimensions of mobility, self-care, usual activities, pain/discomfort, and anxiety/depression at 1-year post-stroke, and this category accounted for 32.45% of all the young and middle-aged stroke population in this study. This indicates that stroke has a significant impact on physical functioning and that boosting recovery from the condition is the most effective approach to increasing RTW rates within 1 year. However, it is worth noting that female patients who were reluctant to work had an EQ-5D index second only to those who were unable to work, which may be related to a higher rate (16.13%) of limitations with self-care. Similarly, male patients who were unable to work retired early and stayed at home full time had lower EQ-5D VAS. This may be due to the fact that male patients are typically the primary breadwinners in their families, their eagerness to RTW is greater, and their self-reported quality of life is lower when they are unable to return to work. Life satisfaction studies indicate that RTW improves health and wellbeing after stroke and is more important than non-RTW for overall life satisfaction. This difference was pronounced for male patients (26, 27).

This research is subject to certain limitations. First, the researcher's classification of the reasons for not returning to work may be subjective, and some patients may report multiple reasons for non-RTW, and for such patients, we ask for the main reason for non-RTW. Second, although 89 of the 435 patients (20.5%) in our study who failed to RTW declared that they left the workforce for reasons of being reluctant to work or to give a reason, we did not have detailed explanations for these decisions. We did not collect information about patient-reported work conditions or job quality, including job stress, job satisfaction, and job safety. Information about patient-reported work conditions, in addition to health and socioeconomic characteristics, is important. This information may help determine patient-centered interventions supporting RTW. Finally, our study included only patients with stroke from a single center, which may caution us from generalizing to a larger population. Furthermore, the sample size available for the study resulted in wide 95% confidence intervals. Larger sample sizes should be considered in future studies to increase the precision of effect estimates.

## 5. Conclusion

More than half of young and middle-aged patients with stroke did not RTW within 1 year. Our study highlights the most frequently cited reasons for non-RTW, how they vary across sociodemographic and clinical profile factors, and their impact on HRQoL at 1 year. In vocational rehabilitation, more focus should be directed to female patients who were reluctant to work and male patients who were full-time homemakers or unemployed.

## Data availability statement

The original contributions presented in the study are included in the article/supplementary material, further inquiries can be directed to the corresponding author.

## Ethics statement

The studies involving human participants were reviewed and approved by the Central Review Committee of the First Affiliated Hospital of Soochow University. The patients/participants provided their written informed consent to participate in this study.

## Author contributions

XP: paper writing and data analysis. ZW: data collection and data organization. LY: data compilation and data analysis. LX: project preparation and thesis revision. All authors contributed to the article and approved the submitted version.
